# A note on estimating absolute cytosolic Ca^2+^ concentration in sensory neurons using a single wavelength Ca^2+^ indicator

**DOI:** 10.1177/17448069241230420

**Published:** 2024-02-21

**Authors:** James P Higham, Ewan St John Smith, David C Bulmer

**Affiliations:** Department of Pharmacology, 2152 University of Cambridge, Cambridge, UK

**Keywords:** Calcium imaging, sensory neurons, fluorescent calcium indicator, Fluo4

## Abstract

Ca^2+^ imaging is frequently used in the investigation of sensory neuronal function and nociception. In vitro imaging of acutely dissociated sensory neurons using membrane-permeant fluorescent Ca^2+^ indicators remains the most common approach to study Ca^2+^ signalling in sensory neurons. Fluo4 is a popular choice of single-wavelength indicator due to its brightness, high affinity for Ca^2+^ and ease of use. However, unlike ratiometric indicators, the emission intensity from single-wavelength indicators can be affected by indicator concentration, optical path length, excitation intensity and detector efficiency. As such, without careful calibration, it can be difficult to draw inferences from differences in the magnitude of Ca^2+^ transients recorded using Fluo4. Here, we show that a method scarcely used in sensory neurophysiology – first proposed by Maravall and colleagues (2000) – can provide reliable estimates of absolute cytosolic Ca^2+^ concentration ([Ca^2+^]_cyt_) in acutely dissociated sensory neurons using Fluo4. This method is straightforward to implement; is applicable to any high-affinity single-wavelength Ca^2+^ indicator with a large dynamic range; and provides estimates of [Ca^2+^]_cyt_ in line with other methods, including ratiometric imaging. Use of this method will improve the granularity of sensory neuron Ca^2+^ imaging data obtained with Fluo4.

## Findings

### Background

Measuring changes in [Ca^2+^]_cyt_ is a commonly used method for determining the sensitivity of sensory neurons to different stimuli and investigating pro-nociceptive signalling pathways, with Fluo4 being a popular choice of single-wavelength Ca^2+^ indicator. As has been discussed in detail previously,^1^ one can estimate [Ca^2+^]_cyt_ from the optical properties of a fluorescent Ca^2+^ indicator using the following equation^2^

(1) $${\left[{Ca}^{2+}\right]}_{cyt}=K_{D}\frac{F-F_{\min}}{F_{\max}-F}$$

Where *K*_D_ is the equilibrium dissociation constant for the binding of Ca^2+^ to the indicator (for Fluo4, this will be taken as 325 nM as estimates vary between 300 nM and 350 nM^1,3,4^); F is the measured emission intensity; F_min_ is the minimum emission intensity (usually found by lysing cells in a high concentration of a Ca^2+^ chelator in Ca^2+^-free bath solution); and F_max_ is the maximum emission intensity (usually found by lysing cells in a high concentration of Ca^2+^). Experimentally, it is difficult and impractical to measure F_min_ and F_max_ for the same set of cells because these measurements require cell lysis, and so F_min_ is often found in a parallel experiment (meaning that measures of F_min_ are made in different cells to those used to measure F and F_max_). This problem can be circumvented if one considers equation (1) in terms of the indicator’s dynamic range (R = F_max_/F_min_), which yields

(2) $${\left[{Ca}^{2+}\right]}_{cyt}=K_{D}\frac{\frac{F}{F_{\max}}-\frac{1}{R}}{1-\frac{F}{F_{\max}}}$$

It is apparent that if R is large, as is the case for Fluo4 (R ≈ 85-100^1^), then 1/R will become negligible (as 1/R << F/F_max_), and equation (2) can be rewritten as

(3) $${\left[{Ca}^{2+}\right]}_{cyt}=K_{D}\frac{\frac{F}{F_{\max}}}{1-\frac{F}{F_{\max}}}$$

The estimate of [Ca^2+^]_cyt_ provided by equation (3) does not require the experimenter to find F_min_. F_max_ can be found easily at the end of each experiment by applying 0.1% Triton-X in a bathing solution containing 10 mM Ca^2+^. As R and K_D_ are properties intrinsic to the indicator, it is not necessary for them to be estimated for each experiment,^1^ though it is important to consider that these parameters are sensitive to the intracellular environment (e.g., pH, ionic composition).^5^ Therefore, adding a straightforward step to the end of each Ca^2+^ imaging experiment can provide an estimate of [Ca^2+^]_cyt_ using Fluo4.

### Estimating changes in [Ca^2+^]_cyt_

**Figure 1.**
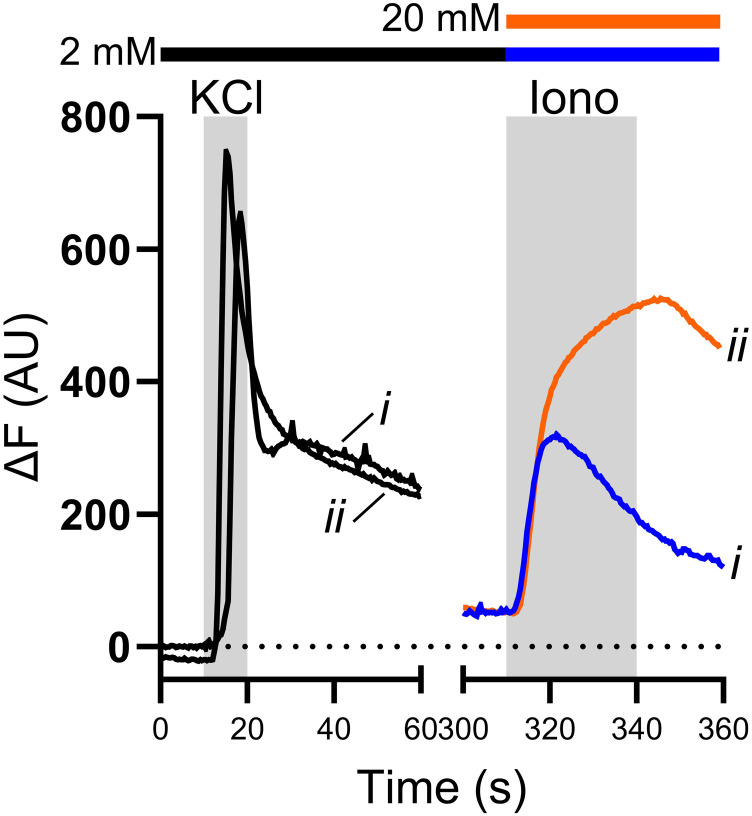
Calibration of Ca^2+^ transients using ionomycin. Example traces showing the change in Fluo4 fluorescence over baseline (ΔF) during the application of 50 mM KCl and 5 µM ionomycin. KCl was applied in the presence of 2 mM bath Ca^2+^ (black traces), and ionomycin was applied in the presence of either 2 mM (blue trace) or 20 mM (orange trace) bath Ca^2+^. In both cases, the peak fluorescence evoked by KCl application was greater than that evoked by ionomycin application, showing that ionomycin application cannot have resulted in saturation of the indicator.

Ionomycin is often used to calibrate Ca^2+^ signals measured in sensory neurons and while this calibration provides some useful information, it cannot be used to estimate absolute [Ca^2+^]_cyt_. This is because ionomycin cannot raise [Ca^2+^]_cyt_ sufficiently to obtain a true F_max_ for the indicator. This is apparent if 50 mM KCl and 5 µM ionomycin are sequentially applied to sensory neurons in a bath solution containing 2 mM Ca^2+^ (Figure 1, trace *i*). Ionomycin application cannot have resulted in saturation of Fluo4 because the magnitude of Ca^2+^ transients evoked by KCl (black traces) exceeded those evoked by ionomycin. Similar results were obtained after raising bath [Ca^2+^] to 20 mM during the application of ionomycin, though the response to ionomycin was greater under these conditions (Figure 1, trace *ii*).

While KCl is useful for identifying viable neurons, its use as a calibration for the magnitude of Ca^2+^ signals in sensory neurons is limited because the magnitude of the response to KCl is dependent on voltage-gated Ca^2+^ channel function and cytosolic Ca^2+^ buffering, which may not be comparable between different sensory neuron subpopulations^6^ or different experimental conditions.

**Figure 2.**
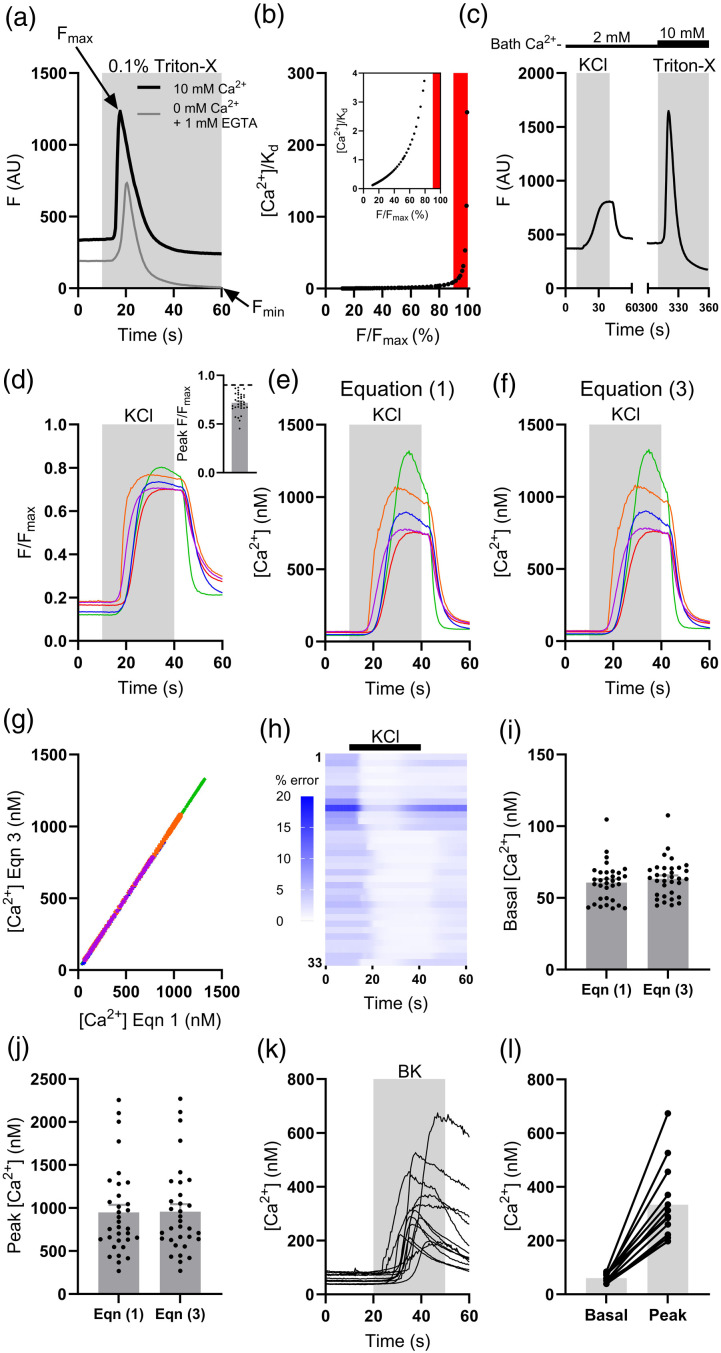
Measuring changes in [Ca^2+^]_cyt_ in sensory neurons using Fluo4. (a) Example traces from two neurons showing the application of 0.1% Triton-X to sensory neurons in the presence of either 10 mM bath Ca^2+^ (black trace) or 0 mM bath Ca^2+^ and 1 mM EGTA (grey trace). The peak of the black trace shows F_max_ for this neuron, while the minimum of the grey trace shows F_min_. The peak of the grey trace shows the liberation of Ca^2+^ from intracellular stores as the neuron is lysed. (b) The relationship between F/F_max_ and [Ca^2+^]/*K*_D_. For F/F_max_ < 90%, [Ca^2+^]/K_D_ remains small and approximately linear (*inset*). However, as F/F_max_ becomes larger (>90%), estimates of [Ca^2+^] become very large and unreliable (red shaded region). (c) Example trace showing uncorrected Fluo4 fluorescence from a single neuron during the application of 50 mM KCl (2 mM bath Ca^2+^) and 0.1% Triton-X (10 mM bath Ca^2+^). (d) Example traces showing Fluo4 fluorescence normalised to F_max_ for five randomly-selected neurons during the application of 50 mM KCl. Traces in (d)-(f) are colour-coded to show data from the same individual neurons. *Inset*: grouped data showing peak F/F_max_ for all neurons imaged (*n* = 33 neurons from three independent DRG preparations, dashed line shows F/F_max_ = 0.9). (e) Example traces showing [Ca^2+^]_cyt_ for five randomly-selected neurons during the application of 50 mM KCl. [Ca^2+^]_cyt_ was calculated from equation (1) using F_min_ = 5.852 AU (from the experiment in (a)). (f) Example traces showing [Ca^2+^]_cyt_ for five randomly-selected neurons during the application of 50 mM KCl. [Ca^2+^]_cyt_ was calculated from equation (3). (g) Scatterplot comparing estimates for [Ca^2+^]_cyt_ calculated using equations (1) and (3) for the five neurons in (d)-(f). Each dataset was fit with a straight line (R^2^ > 0.99); the average slope was 1.006 ± 0.0004 and each line passed approximately through the origin, showing a high congruence in the estimates of [Ca^2+^]_cyt_ provided by equations (1) and (3). (h) Heatmap showing the percentage error in the estimate of [Ca^2+^]_cyt_ between equations (1) and (3) for each neuron (black bar shows KCl application). (i) Grouped data showing the average baseline [Ca^2+^]_cyt_ calculated using equations (1) and (3) for each neuron (*n* = 33 neurons from three independent DRG preparations). Data analysed using a Mann-Whitney U-test. (j) Grouped data showing the peak [Ca^2+^]_cyt_ calculated using equations (1) and (3) for each neuron (*n* = 33 neurons from three independent DRG preparations). Data analysed using a Mann-Whitney U-test. (k) Example traces showing [Ca^2+^]_cyt_ (calculated using equation (3)) during the application of 250 nM BK (n = 13 neurons from two independent DRG preparations). (L) Grouped data showing average basal (pre-BK; mean of first 10 s of recording) and peak (post-BK) [Ca^2+^]_cyt_ (n = 13 neurons from two independent DRG preparations).

The absolute F_min_ or F_max_ for Fluo4 – as with other indicators – can be found by applying 0.1% Triton-X (to lyse cells) with either a high concentration of a Ca^2+^ chelator (1 mM EGTA) in Ca^2+^-free bathing solution (Figure 2(a), grey trace), or 10 mM Ca^2+^-containing bathing solution (Figure 2(a), black trace), respectively. The transient increase in F following the application of Triton-X/EGTA in Ca^2+^-free bathing solution (Figure 2(a), grey trace) partly represents liberation of Ca^2+^ from intracellular stores. It is important to note that in the case of equation (3), as F tends towards F_max_, 1 – F/F_max_ will tend towards zero and, hence, estimates of [Ca^2+^]_cyt_ become unreasonably large. While F/F_max_ < 90%, F/F_max_ is approximately linear with [Ca^2+^]_cyt_ and provides a reliable measure of [Ca^2+^]_cyt_ (Figure 2(b)). Beyond 90%, F/F_max_ no longer provides a reliable metric of [Ca^2+^]_cyt_ and estimates become unreasonably large – as such, this has been dubbed the “zone of unreliability” (Figure 2(b), red shaded region^7^). It is, therefore, important to ensure that F measured during an experiment remains within the approximately linear region of equation (3) and does not exceed 90% F/F_max_.

To test the utility of equation (3) for measuring [Ca^2+^]_cyt_ in sensory neurons, 50 mM KCl was used to induce depolarisation-dependent Ca^2+^ transients (*n* = 33 neurons, 2 mM bath Ca^2+^, Figure 2(c)). Estimates of [Ca^2+^]_cyt_ calculated using equations (1) and (3) were compared; the value for F_min_ used in equation (1) was found in the experiment shown in Figure 2(a). F_max_ was found for each neuron at the end of each experiment. The peak F evoked by KCl application remained below 90% of that evoked by Triton-X/10 mM Ca^2+^; peak F/F_max_ was 0.72 ± 0.02 (range: 0.45-0.87, Figure 2(d)). As such, reliable estimates of [Ca^2+^]_cyt_ were possible. Figure 2(e) and (f) show example traces of [Ca^2+^]_cyt_ obtained using equations (1) and (3), respectively. The data look qualitatively very similar. The congruence in the estimates of [Ca^2+^]_cyt_ found using equations (1) and (3) (Figure 2(g)) demonstrates that the dynamic range of Fluo4 is of sufficient magnitude to make its reciprocal negligibly small relative to F/F_max_. The percentage error in the estimates of [Ca^2+^]_cyt_ provided by equations (1) and (3) was lowest during the peak of the KCl-evoked Ca^2+^ transient (0.97 ± 0.06%) and highest during baseline (4.36 ± 0.43%, Figure 2(h)). No difference in the estimated average baseline [Ca^2+^]_cyt_ was found between equations (1) and (3) (*p* = .26, Figure 2(i)). The estimates of peak [Ca^2+^]_cyt_ provided by equations (1) and (3) were also no different (*p* = .78, Figure 2(j)). Importantly, estimates calculated from equation (3) of both baseline (63.4 ± 2.3 nM) and peak (956.9 ± 88.1 nM) [Ca^2+^]_cyt_ agreed with previous estimates made using ratiometric Ca^2+^ imaging.^8–11^ Changes in [Ca^2+^]_cyt_ evoked by 50 mM KCl are relatively large, but this method can also be used to calibrate smaller changes in [Ca^2+^]_cyt_. The application of the algogenic mediator bradykinin (BK, 250 nM, 2 mM bath Ca^2+^) to sensory neurons raised [Ca^2+^]_cyt_ – calculated using equation (3) – from 60.3 ± 4.8 nM to 333.3 ± 39.6 nM (*n* = 13 neurons, Figure 2(k) and (l)).

### Basal [Ca^2+^]_cyt_

**Figure 3.**
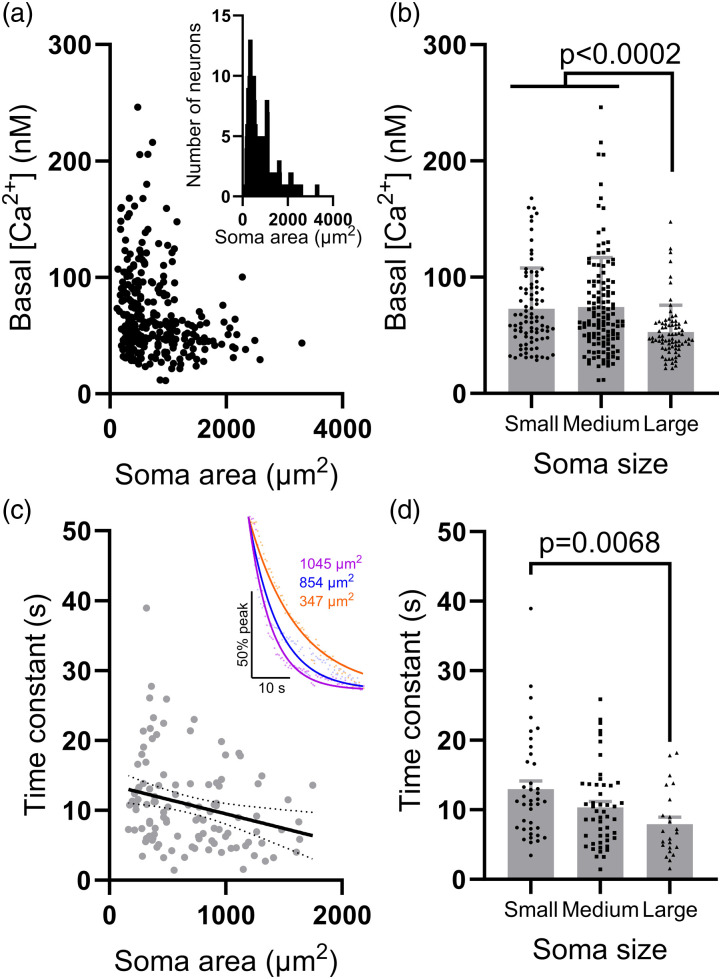
Measuring basal [Ca^2+^]_cyt_ in sensory neurons parsed by soma size. (a) Scatterplot showing soma area and average basal [Ca^2+^]_cyt_ (*n* = 293 neurons from three independent DRG preparations). *Inset*: frequency distribution of soma sizes. (b) Grouped data showing the average basal [Ca^2+^]_cyt_ for small, medium and large sensory neuronal soma. Data analysed using a Kruskal-Wallis ANOVA with Dunn’s post-hoc tests. (c) Scatterplot showing soma area versus time constant for the decay of KCl-evoked Ca^2+^ transients (*n* = 112 neurons from three independent DRG preparations). Line of best fit shown to illustrate modest negative correlation (solid black line with 95% confidence limits shown as dashed lines). *Inset*: traces showing the decay of KCl-evoked Ca^2+^ transients in exemplar small- (orange), medium- (blue), and large-area (purple) neurons. (d) Grouped data showing the time constant for KCl-evoked Ca^2+^ transients in small, medium and large sensory neuronal soma. Data analysed using a Kruskal-Wallis ANOVA with Dunn’s post-hoc tests.

Multiple factors, including ageing^11^ and inflammation,^12^ can affect basal [Ca^2+^]_cyt_ in rodent sensory neurons. We have examined the effect of soma size on resting [Ca^2+^]_cyt_ in sensory neurons (Figure 3(a); soma area distribution shown in *inset*). Neurons were parsed into subgroups by soma area (A): small (A <400 µm^2^, *n* = 86), medium (400 < A <1000 µm^2^, *n* = 129) and large (A >1000 µm^2^, *n* = 78). Although there was no difference in resting [Ca^2+^]_cyt_ between small- and medium-area neurons (73.0 ± 3.8 nM vs 74.3 ± 3.8 nM, *p* > .99, Figure 3(b)), basal [Ca^2+^]_cyt_ in large-area neurons (52.8 ± 2.6 nM) was lower than both small- (*p* = .0002) and medium-area (*p* < .0001) neurons (Figure 3(b)). Average basal [Ca^2+^]_cyt_ across all neurons in this sample was 68.2 ± 2.2 nM (*n* = 293 neurons from three independent DRG preparations), in agreement with previous estimates in rodent sensory neurons.^8–14^ A modestly reduced resting [Ca^2+^]_cyt_ in large-diameter sensory neurons has been observed in rat,^6^ but corroborating observations in mouse sensory neurons are lacking. It is not immediately clear why large-area sensory neurons would exhibit a lower resting [Ca^2+^]_cyt_, though it could be due to more efficient [Ca^2+^] buffering. To test this possibility, another sample of neurons (*n* = 112 neurons from three independent DRG preparations) was stimulated with 50 mM KCl and the decay of evoked Ca^2+^ transients (expressed as the time constant for the decay of the transient) was analysed across different neuronal sizes. There was a modest negative correlation between soma area and time constant (r = −0.26, *p* = .0061, Figure 3(c)). KCl-evoked Ca^2+^ transients in small-area neurons exhibited a time constant of 13.0 ± 1.2 s (*n* = 39), compared to 8.0 ± 1.0 s (*n* = 23) in large-area neurons (*p* = .0068, Figure 3(d)). KCl-evoked Ca^2+^ transients in medium-area neurons exhibited an intermediate time constant (10.4 ± 0.8 s, *n* = 50) which was indistinguishable from that in small- (*p* = .25) and large-area (*p* = .26) neurons (Figure 3(d)). The more rapid decay of KCl-evoked Ca^2+^ transients in large-area neurons (compared with small-area neurons) is consistent with a greater capacity for Ca^2+^ buffering.^6^

In summary, the method put forward by Maravall and colleagues^1^ provides a straightforward, easy-to-implement and – importantly – reliable protocol for estimating [Ca^2+^]_cyt_ in sensory neurons using a high dynamic range single-wavelength indicator without the need to estimate F_min_. Estimates of [Ca^2+^]_cyt_ in sensory neurons made using this method with Fluo4 are well in-line with estimates made using other methods, including ratiometric Ca^2+^ imaging. F_min_ need not be estimated because the dynamic range of Fluo4 is sufficiently large, as evidenced by the close alignment of estimates of [Ca^2+^]_cyt_ between equations (1) and (3). Use of this method broadens the utility of Fluo4 and will enable more rigorous quantification of Ca^2+^ signalling in sensory neurons using Fluo4.

## Methods

### Preparation of sensory neurons

All animal work was carried out in accordance with the Animals (Scientific Procedures) Act 1986. Mice (C57Bl/6, Charles River) were housed in groups of up to six littermates under a 12 h light/dark cycle with bedding material, enrichment (e.g., igloos, tunnels, etc) and ad libitum access to food and water. All mice used were male aged 8-14 weeks.

Sensory neurons were prepared as described previously.^15–17^ Briefly, dorsal root ganglia (DRG, T12-L5) were removed from the spinal column and enzymatically digested in collagenase (1 mg/mL) and trypsin (1 mg/mL). DRG were then mechanically dispersed by trituration through a pipette tip. Dispersed neurons were seeded onto glass-bottomed culture dishes coated with poly-d-lysine and laminin (MatTek, MA, USA). Neurons were incubated with supplemented L-15 growth medium (10% foetal bovine serum, 2.6% NaHCO_3_, 1.5% d-glucose and 2% penicillin/streptomycin) at 37°C in 5% CO_2_ and used for Ca^2+^ imaging no more than 24 h after plating.

### Ca^2+^ imaging

Growth medium was aspirated from culture dishes and neurons were incubated with 10 µM Fluo4-AM for 30-45 min at room temperature (shielded from light). Neurons were then washed and bathed in extracellular bath solution containing (in mM): 140 NaCl, 4 KCl, 2 CaCl_2_, 1 MgCl_2_, 4 d-glucose, 10 HEPES (pH 7.35-7.45 with NaOH; 290-310 mOsm). Ca^2+^-free bath solution was identical except for the omission of CaCl_2_ and that it contained 1 mM EGTA and 2 mM MgCl_2_. Dishes were mounted on an inverted Nikon Eclipse TE-2000S microscope and cells were visualised under brightfield illumination with a 10x air objective. Neurons were superfused with bath solution via a flexible inflow tube (placed adjacent to cells of interest) (AutoMate Scientific, CA, USA) fed by a gravity-fed perfusion system (Warner Instruments, CT, USA).

All imaging was carried out at room temperature. Images were acquired at 2.5 fps with 100 ms exposure using a Retiga Electro CCD camera (QImaging, BC, Canada). Fluo4 was excited by a 470 nm light source (Cairn Research, UK) and emission at 520 nm was recorded using µManager.^18^ For experiments using 50 mM KCl as a stimulus, KCl was superfused for 30 s following a 10 s baseline. For experiments using 250 nM bradykinin as a stimulus, bradykinin was superfused for 30 s following a 20 s baseline. At the end of all experiments, 0.1% Triton-X in 10 mM Ca^2+^ bath solution was superfused to lyse cells and yield an estimate for F_max_ for each neuron. F_min_ was estimated in one experiment by applying 0.1% Triton-X to neurons bathed in Ca^2+^-free bath solution containing 1 mM EGTA.

### Data analysis

Regions of interest were manually traced around neurons and the average pixel intensity per region per frame was calculated using ImageJ. After the subtraction of background fluorescence, measured fluorescence values for each neuron were calibrated to [Ca^2+^] using equations (1) and (3) using a *K*_D_ for Fluo4 of 325 nM.

Datasets were scrutinised to ensure that they met the assumptions of parametric analyses (normality tested using the Shapiro-Wilk test; equality of variances tested using F-tests) and, where appropriate, non-parametric, rank-based alternatives were used. Details of statistical tests used are in the figure legends. To find the time constant for the decay of KCl-evoked Ca^2+^ transients, data were fit with a one-phase exponential decay. The time constant gives the time for the transient to decay by a factor of 1/*e*, that is, ∼37% of peak. This is equivalent to T_50_/*ln*(2), where T_50_ is the half-life of the decay of the transient, that is, the time for the transient to decay by a factor of 1/2. Analysis was carried out in GraphPad Prism (GraphPad Inc.). Grouped data are displayed as mean ± standard error.
